# Bond strength according to the moment of fiber post cutting fixed with self-adhesive cement to the root dentin 

**DOI:** 10.4317/jced.57671

**Published:** 2021-01-01

**Authors:** Emerson Silva, Carolina Pereira, Francisco Limeira, Pollyanna Affonso, Allyson Moreira, Cláudia Magalhães

**Affiliations:** 1 Post-Graduate Program in Dentistry, Universidade Federal de Minas Gerais, Belo Horizonte, Brazil; 2 Professor, Department of Restorative Dentistry, Faculty of Dentistry, Universidade Federal de Minas Gerais, Belo Horizonte, Minas Gerais, Brazil; 3 Professor, Dentistry graduation, Faculty President Antônio Carlos, Teófilo Otoni, Minas Gerais, Brazil; 4 Undergraduate student, Faculty of Dentistry, Universidade Federal de Minas Gerais, Belo Horizonte, Minas Gerais, Brazil

## Abstract

**Background:**

The fiber posts require a cut in the coronal portion to adjust it to the available clinical space. The cutting of posts cemented may generate tension via bur vibrations of drill on the bonding interface, with the possibility of decreasing the bond strength. Thus, this study aimed to evaluate if the moment of cutting the fiber posts has an effect on its bond strength when fixed with self-adhesive resin cement.

**Material and Methods:**

Thirty-six bovine teeth were randomly divided into three groups after endodontic treatment and post space preparation (n = 12): IAC- the fiber posts were cutting immediately after cementation; ACR - the fiber posts were cutting after coronal reconstruction with resin; Control - the fiber posts were not cut. The fiber posts were cemented with self-adhesive cement (RelyX U200 - 3M ESPE). After 24 h, the teeth were sectioned perpendicularly and the push-out test was performed in a universal testing machine at a speed of 0.5 mm/min, until failure. Data were analyzed using two-way ANOVA and Tukey’s post-hoc test.

**Results:**

The effect of moment of fiber posts cutting (*p* = 0.44) and the interaction between the factors moment of post cutting and third root (*p* = 0.81) had no significant. The root third factor showed a significant effect (*p* = 0.01). The bond strength in the apical third was significantly lower than in the middle (*p* = 0.00) and coronal (*p* = 0.01) thirds.

**Conclusions:**

The moment of post cutting has no effect on the bond strength of fiber posts fixed with self-adhesive cement to the root canal.

** Key words:**Fiber post, self-adhesive resin cement, push-out bond strength, operative dentistry.

## Introduction

With the increase in demand for aesthetic restorations, endodontically treated teeth with little coronal structure have been restored using fiber posts (1,2). The advantages of fiber posts are related to their mechanical properties, including an elastic modulus which is close to dentin, high flexural strength, compatibility with Bis-GMA-based resin, biocompatibility and resistance to corrosion (3-5).

However, the success of these intraradicular restorations is dependent upon adequate bonding at the post-dentin interface (1). When the post is luted to the root canal, two interfaces occur. One of them is between the post and the cement, and the other is between the cement and the root dentin (6). Most commonly reported mode of clinical failure in fiber post is debonding from root dentin (7). The main problem that is believed to adversely affect the bonding strength of fiber post is the blockage of cement adhesion by obstruction of the dentinal tubules by the Gutta-percha and sealer remnants, dentine debris and smear layer (8). In addition to external factors such as irrigants, types of adhesives, and endodontic sealers, dentin-related factors such as dentin status and orientation of dentin tubules also affect these interfaces (9).

Normally, fiber posts cementation involves the use of conventional resin cements associated with adhesive systems (10). However, conventional resin cement has high technique sensitivity (11). Thus, the self-adhesive resin cements have been developed to minimize this problem, as an alternative to the cementation process, eliminating any pretreatment of the dentin (1,11). The self-adhesive resin cement has primary adhesion mechanism based on the micromechanical retention and chemical interaction between the monomer acidic groups and hydroxyapatite. The multifunctional monomers with phosphoric acid groups simultaneously demineralize and infiltrate enamel and dentin (12).

Upon post-cementation, the rehabilitation of the coronal portion requires a core buildup that bonds to the radicular portion and creates a substrate for a total or partial crown (13,14). The fiber posts are available in standardized lengths and require a cut in the coronal portion to adjust it to the available clinical space. Post sectioning can be performed prior to post insertion into the root canal, immediately after post cementation or after coronal portion reconstruction. The cutting of posts cemented may generate tension via bur vibrations of drill on the bonding interface, with the possibility of decreasing the bond strength and compromising the longevity of the post-retained restoration (15).

Thus, this study aimed to evaluate if the moment of cutting the fiber posts has an effect on its bond strength when fixed with self-adhesive resin cement. The null hypothesis tested was: the moment when post cutting occurs does not affect the bond strength of the fiber posts fixed with self-adhesive cement.

## Material and Methods

-Sample Selection and Root Canal Preparation 

Thirty-six bovine teeth with similar dimensions and without structural defects were selected. The teeth were cleaned, disinfected in 0.1% thymol solution for 1 day, and kept frozen until preparation. The crowns were sectioned at the cementoenamel junction using a diamond disk (KG Sorensen, Cotia, SP, BR) in a low-speed handpiece under air/water cooling. The roots length was standardized at 19.0 mm.

In accordance with the Arouca Law (nº 11,794 of October 8, 2008), adopted by the Ethics Committee on the Use of Animals, of the Federal University of Minas Gerais, this study does not need to be submitted to this Committee, as it only uses the teeth of the animal slaughtered in a slaughterhouse certified by the Sanitary Surveillance.

The root canals were instrumented with rotary files (ProDesign System, Easy, Belo Horizonte, MG, BR) and irrigated between each filing with 2.5% NaOCl (Asfer Indústria Química; São Caetano do Sul, SP, BR). The smear layer was removed with 2 ml of 17% ethylenediaminetetraacetic acid (Biodinâmica, Ibiporã, PR, BR) for 3 min. After aspiration, absorbent paper points (Dentisply Maillefer, Ballaigues, SWZ) were used to dry the canal walls. The root canals were filled with gutta percha points (Dentisply Maillefer, Ballaigues, SWZ) using the cold lateral condensation technique and sealed with an epoxy resin-based sealer (AH Plus, Dentisply Maillefer, Ballaigues, SWZ). Next, the roots were storage in 100% humidity at 37°C for 7 days.

The external surfaces of the roots were dipped in melted wax, resulting in a 0.2- to 0.3-mm thick wax layer, to simulate the periodontal ligament. Then, the wax-covered roots were included in polyvinyl chloride cylinders (19,05 mm) and embedded with chemically polymerizable resin, without exotherm (Liqui Vidro, Alpha Resiqualy Ind. and Com. de Resinas Ltda., São Paulo, SP, BR). After inclusion, the roots were storage in 100% humidity at 37°C for 48 hours.

-Fiber Post Cementation and Cutting 

The post space was prepared with Largo burs #3 to #5 as recommended by the post manufacturer (Reforpost #3, Angelus, Londrina, PR, BR). The post space length was 14 mm, maintaining 4-mm apical filling material. After preparation, the root dentin was cleaned with 10 ml of distilled water and drying with absorbent paper points. The upper surface of the cylinders was covered with black adhesive tape to protect the roots them from external light exposure. The roots were then randomly distributed into three groups (n = 12): IAC- the fiber posts were cutting immediately after cementation; ACR - the fiber posts were cutting after coronal reconstruction with resin; Control - the fiber posts were not cut.

The fiber posts were cleaned with 37% phosphoric acid (Condac 37, FGM, Joiville, SC, BR) for 1 min, rinsed with distilled water for one minute and dried with compressed air. One layer of silane was actively applied for 1 min on the post surface, followed by dried with a jet of hot air for 1 min.

The resin cement RelyX U200 (3M ESPE, St Paul, MN, EUA) was mixed and inserted into the canal with a syringe and a needle tip. Subsequently, the fiber post was positioned and light activated for 30 s in each position (buccal and lingual) with a polywave LED light-polymerizing unit (LED Bluephase, Ivoclar-Vivadent, SC, FL), wavelength range of 440-480 nm and 1340 mW/cm2.

For IAC group, the fiber posts were cuting immediately after photoactivation of the resin cement using a #3098 diamond tip (KG Sorensen, Cotia, SP, BR), at 5 mm from the cervical edge, operated by a high-speed hand-piece and copious water spray.

For the ACR group, coronary reconstruction was performed using Filtek Z-350 composite (3M ESPE, St Paul, MN, EUA) before the cutting procedure. To standardize the core, were used Polyvinyl Chloride matrices for lower incisors (Transparent Crowns, TDV Dental Ltda, Pomerode, SC, BR). The fiber posts were sectioned to the same level of the resin core, using a #3098 diamond tip (KG Sorensen, Cotia, SP, BR), with copious water spray. All specimens were stored in a moist environment at 37°C for 24 hours before being prepared for mechanical testing.

-Push-out Bond Strength Test

The roots were fixed in acrylic plates and sectioned transversely with a precision saw (IsoMet 1000, Buehler, Lake Bluff, IL, USA) to obtain 2 specimens of each root third with a thickness of 1.0 mm. The thickness of the specimens was measured with a digital caliper (Mitutoyo Series 500, Suzano, SP, BR). The test were performed in each of the sections by using a universal testing machine (Emic DL 3000, Emic, São José dos Pinhais, PR, BR) with a load cell of 200 N and operating at a speed of 0.5 mm/min. The load was applied from the most apical surface in the coronal direction. The maximum bond strength was obtained by measuring the displacement of the fiber post and resin cement from the root canal. To express the bond strength in MPa, the load obtained in N was divided by the area of the bonded interface, which was calculated by using the following equation: A = 2πrh, where π is 3.14, r is the radius of the post (measured with a stereomicroscope) and h is the slice thickness in millimeters.

-Failure Mode Analysis

After the push-out test, each specimen was observed and photographed using a stereomicroscope (Stereo Discovery. V8, Carl Zeiss AG, Oberkochen, GER) at ×50 magnification to determine the failure modes. Two calibrated examiners (kappa = 0.84) independently evaluated the failure mode, classificated as: 1) adhesive between dentin and cement; 2) adhesive between post and cement; 3) mixed, part of the cement adhered in the dentin and the pin 3); cohesive in cement; and 5) cohesive in dentin. Disagreements between the examiners were resolved by consensus.

-Statistical Analysis

Normal distribution and homogeneity of variances of the push-out bond strength were tested using the Shapiro-Wilk (*p* > 0.05) and Levene tests (*p* > 0.05), respectively. The effects of the moment of post cutting and third root, as well as the interaction of these factors in the mean bond strength, were verified by 2-way ANOVA and Tukey’s honest significant differences post-hoc test. All statistical analyses were performed using the statistical software SPSS v. 20 (IBM; Armonk, NY, USA) at a 5% level of significance.

## Results

The results of the 2-way ANOVA showed that the effect of moment of fiber posts cutting (*p* = 0.44) and the interaction between the factors moment of post cutting and third root (*p* = 0.81) had no significant. The root third factor showed a significant effect (*p* = 0.01). The Tukey test demonstrated that the bond strength in the apical third was significantly lower than in the middle (*p* = 0.00) and coronal (*p* = 0.01) thirds. There was no significant difference in bond strength between the middle and coronal thirds (*p* = 0.84) (Table 1).

**Table 1:**
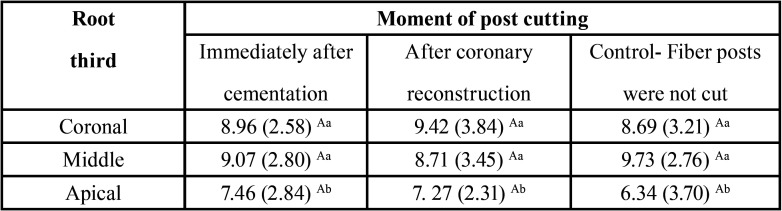
Mean push-out bond strength (±SD) of experimental groups according to moment of post cutting and root third.

The failure mode frequency is shown in Table 2. The failure in interface dentin-cement was the mode most frequent, irrespective of moment of post cutting and root third.

**Table 2:**
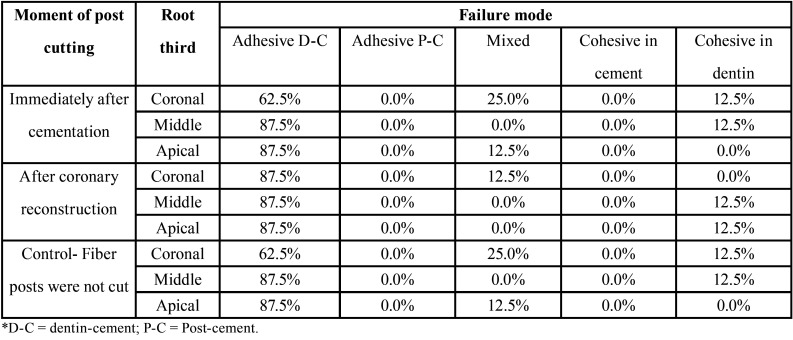
Distribution of failure modes according to moment of post cutting and root third.

## Discussion

The bond strength of fiber posts to the root canal has been the subject of several studies evaluating adhesive materials and cementation protocols (2,16,17,18). However, there is a gap regarding the orientation of the ideal moment for the execution of the cutting of fiber posts when using of post-retained restoration in restored procedures. The results of this study showed that the moment of fiber posts cutting did not affect the bond strength of posts cemented with self-adhesive resin in the root canal, leading to the acceptance of the null hypothesis.

Our results are in agreement with the other study (15), the only that had previously analyzed the effect of the moment of post cutting on the bond strength values. This study also showed that the moment of fiber post cutting not affected the bond strength when auto-adhesive resin cement was used. Thus, the results of both studies contradicted the perspective that the vibration during cutting generated a stress capable of reducing the bond strength, especially when the post was sectioned immediately after cementation. As well as, the concept that the reconstruction of the coronal portion would prevent the transmission of the stress generated by the vibration of the drill and maintains the integrity of the adhesive interface (15).

The absence of effect of the moment of the post cutting on the bond strength values can be explained in part by the use of self-adhesive resin cement. Borges *et al.* (15) observed that the post cutting immediately after cementation resulted in the lowest bond strength values just when conventional resin cement was used. Thus, these same authors assume that chemical bonding produced by self-adhesive resin cement results in a more sTable bonding interface. Self-adhesive resin cements present a less sensitive technique because they eliminate the precementation steps of the resin cement inside the root canal (19). Moreover, these cements promote better bond strength results than conventional resin cements (20-22).

The root third factor had a significant effect on bond strength. Although the self-adhesive resin cement is a dual-polymerized material, the apical third had the lowest bond strength values. This result could be related to the higher stress concentration of the apical slice due the higher porosity on apical region because the difficulty of the resin cement insertion and the possibility of the air confined during post insertion (23). High polymerization shrinkage stresses have been reported in resin cements because of the high C-factor in the root canal (24). Under shrinkage effect, gaps occur when those stresses are higher than bond on adhesive interface. Also, light intensity that reaches the cervical and middle thirds provided higher bond strengths to dentin (25). Limited light-transmitting ability of fiber posts can reflect in a reduction of the resin cement conversion degree with increased length of simulated root canals (12).

The most prevalent failure mode observed in this study reinforce the statement that the most sensible interface is between dentin and resin cement. It is the interface where the higher stress concentration is located. The push-out test presents somewhat similar characteristics in terms of effect from forces when under clinical service on the fiber post, interfaces, and root dentin, that is, vectors of shear stress inducing pull-out of fiber post (17). These findings suggest that the priority is to improve adhesion of resin cement to dentin considering the context evaluated in our study.

Considering the experimental conditions employed in this study, the moment of the post cutting did not have an effect on the bond strength. Thus, the dentist must determine the most convenient time to cut the post, considering the ease of handling it during cementation, the need to keep the operative field clean and dry and the procedures used for coronary reconstruction.

## Conclusions

The results indicate that the moment of post cutting has no effect on the bond strength of fiber posts fixed with self-adhesive cement to the root canal. However, it is important to emphasize that just the immediate bond strength of self-adhesive resin cement was evaluated. Further studies with additional resin cements and aged samples are required.
